# Quantifying Migration Behaviour Using Net Squared Displacement Approach: Clarifications and Caveats

**DOI:** 10.1371/journal.pone.0149594

**Published:** 2016-03-03

**Authors:** Navinder J. Singh, Andrew M. Allen, Göran Ericsson

**Affiliations:** Department of Wildlife, Fish and Environmental Studies, Swedish University of Agricultural Sciences, Umeå, SE-90183, Sweden; Deakin University, AUSTRALIA

## Abstract

Estimating migration parameters of individuals and populations is vital for their conservation and management. Studies on animal movements and migration often depend upon location data from tracked animals and it is important that such data are appropriately analyzed for reliable estimates of migration and effective management of moving animals. The Net Squared Displacement (NSD) approach for modelling animal movement is being increasingly used as it can objectively quantify migration characteristics and separate different types of movements from migration. However, the ability of NSD to properly classify the movement patterns of individuals has been criticized and issues related to study design arise with respect to starting locations of the data/animals, data sampling regime and extent of movement of species. We address the issues raised over NSD using tracking data from 319 moose (*Alces alces*) in Sweden. Moose is an ideal species to test this approach, as it can be sedentary, nomadic, dispersing or migratory and individuals vary in their extent, timing and duration of migration. We propose a two-step process of using the NSD approach by first classifying movement modes using mean squared displacement (MSD) instead of NSD and then estimating the extent, duration and timing of migration using NSD. We show that the NSD approach is robust to the choice of starting dates except when the start date occurs during the migratory phase. We also show that the starting location of the animal has a marginal influence on the correct quantification of migration characteristics. The number of locations per day (1–48) did not significantly affect the performance of non-linear mixed effects models, which correctly distinguished migration from other movement types, however, high-resolution data had a significant negative influence on estimates for the timing of migrations. The extent of movement, however, had an effect on the classification of movements, and individuals undertaking short- distance migrations can be misclassified as other movements such as sedentary or nomadic. Our study raises important considerations for designing, analysing and interpreting movement ecology studies, and how these should be determined by the biology of the species and the ecological and conservation questions in focus.

## Introduction

The field of movement ecology has expanded rapidly in the last three decades across terrestrial and aquatic environments, largely attributed to the vast improvements in data collection techniques and advancements in theory and analytical methods [1,2]. Generally, for moving animals, scientists, conservationists, managers and policy makers are interested in knowing ‘why they move’, ‘when’, ‘where to’ and ‘how fast or slow’[3–7]. However, aspects such as the extent of movement of a species, frequency and consistency of the data, do not render easy analyses and interpretation, given that statistical methods have also advanced rapidly and work under specific assumptions depending upon the biological questions. Under these circumstances, it is important to evaluate the effects of study design of data collection on movement analyses. This will have important implications for both ecological and conservation related conclusions on movements [8–11].

**Fig 1:**
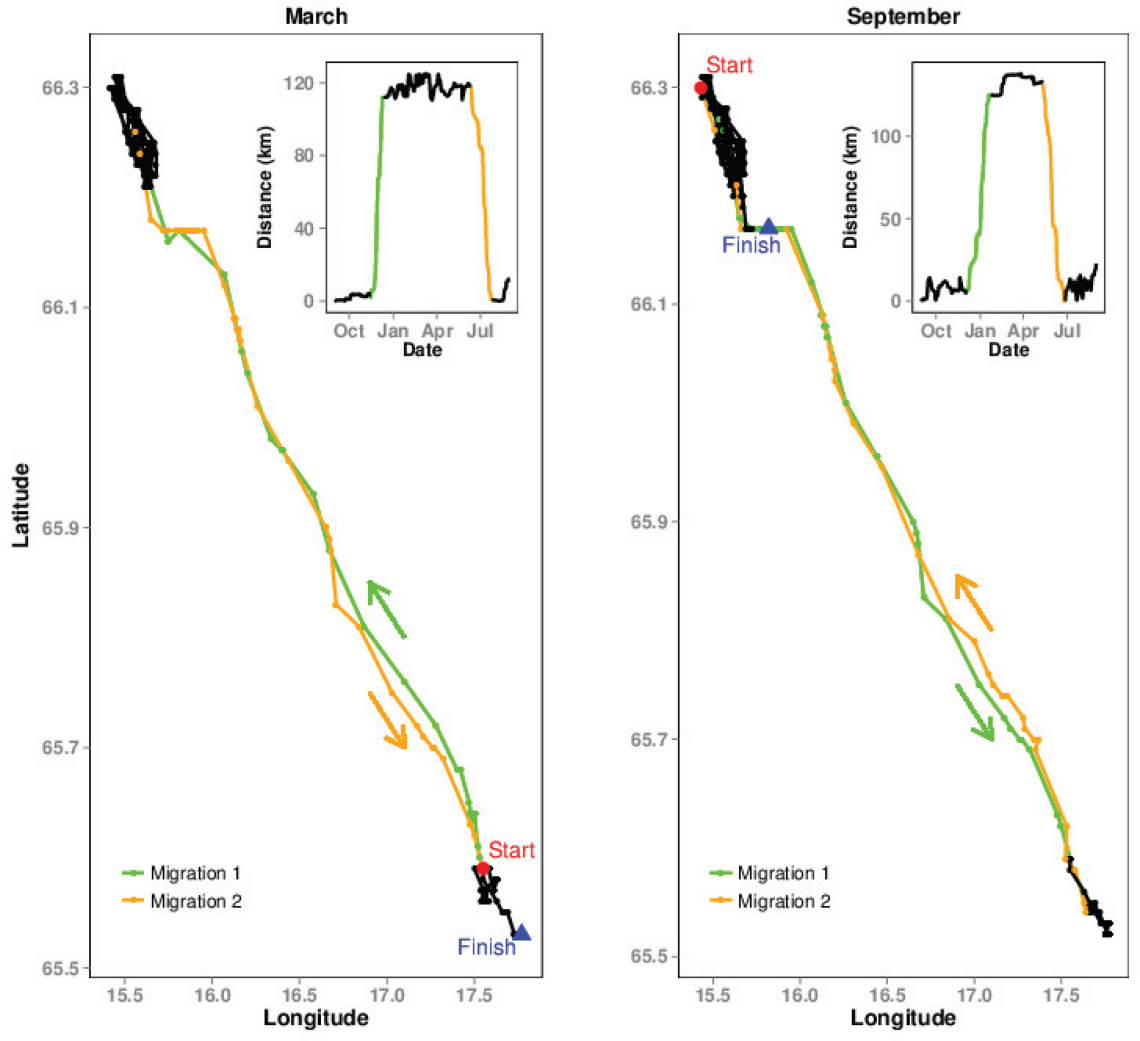
Migration 1 and Migration 2 explanation. Example of a migration path of an individual moose using a start date of either March (left) or September (right). In March, Migration 1 (the first leg) is the spring migration, however, when the start date is September, Migration 1 is the autumn migration. In both Figs the start and end of migration were estimated by fitting a migratory model to the net squared displacement (see Table 1) for details.

Migration is one of the most studied movement behaviours and the first vital question that migration ecologists generally encounter is how to quantitatively distinguish between migration and other types of movements that individuals exhibit. This is important in order to identify what proportion of a population migrates and which conservation/management strategies can be employed for different parts of the population [12,13]. Some quantitative studies have approached this aspect by defining individuals as migratory when they move between breeding and wintering areas in spring and autumn [14], when the seasonal ranges are exclusive [15], when seasonal ranges are a specified distance apart [16], or based on political boundaries depending upon when individuals reached a certain site [14]. Advanced movement modelling approaches can be used to categorize movements, such as state-space models and continuous time movement models [17–21]. These methods may be used to identify different behavioural modes along the movement path, such as foraging or migratory movements [20,22]. Here we focus on a recently developed method called the ‘Net Squared Displacement (NSD) approach’ that combines the displacement of an individual with non-linear mixed effects models in order to distinguish between movement strategies of migration, range residency, nomadism and dispersal [23–25]. Migration in this approach is defined as a double sigmoid or s-shaped function, which is repeated within a year, leading to an exact return to the departure location, therefore resembling a non-linear curve (see Table 1 and Fig 1). With the onset of migration, the net squared displacement from the starting location increases, till it reaches a plateau i.e the other seasonal range. As the individual returns to the original location, the net squared displacement decreases and reaches zero (Fig 1). This approach, which has become popular, follows the more traditional views of migration, consisting of seasonal movements between summer and winter ranges with an approximate return to the departure locations (Fig 1).

**Table 1:** Mathematical equations and the description of parameters used in the Net Squared Displacement movement models in the study. Concordance criterion is used to estimate the model fits.

Model	Equation	Description
NSD model for migration	$NSD=\frac{\delta }{1+exp\left(\frac{\theta_{s}-t}{\varphi_{s}}\right)}+\frac{-\delta }{1+exp\left(\frac{\theta_{a}-t}{\varphi_{a}}\right)}$	distance (δ), timing (θs—spring and θa—autumn) and duration of spring (φs) and autumn (φa) migrations
NSD model for mixed migration	$NSD=\frac{\delta_{s}}{1+exp\left(\frac{\theta_{s}-t}{\varphi_{s}}\right)}+\frac{-\delta_{a}}{1+exp\left(\frac{\theta_{a}-t}{\varphi_{a}}\right)}$	Same as migration except δ can vary according to spring and autumn migrations
NSD model for dispersal	$NSD=\frac{\delta }{1+exp\left(\frac{\theta -t}{\varphi }\right)}$	Where δ is the asymptotic height, θ is the time at which dispersal reaches half δ, φ is the time between reaching half and ¾ of dispersal, and *t* is the number of days since first location
NSD model for home range	*NSD* = ∅_1_[1 − *exp*(∅_2_*t*)]	Where ∅_1_ is the asymptote at the steady-state equilibrium and ∅_2_ is the logarithm of the rate constant
NSD model for nomadism	*NSD* = 4 * *D* * *t*	Single-parameter linear regression model, where D is the diffusion constant (multiplied by 4 for two-dimensional movements) and t denotes the time since start
Concordance Criterion	${CC}_{i}=\frac{\sum_{j=1}^{n_{i}}{\left(y_{ij-{\hat{y}}_{ij}}\right)}^{2}}{\sum_{j=1}^{n_{i}}{\left(y_{ij}-\bar{y}\right)}^{2}+\sum_{j=1}^{n_{i}}{\left({\hat{y}}_{ij}-\bar{\hat{y}}\right)}^{2}+\;n_{i}{\left(\bar{y}-\bar{\hat{y}}\right)}^{2}}$	Here $\bar{y}$ and $\hat{y}$ are the means of the observed (y_ij_) and predicted (${\hat{y}}_{ij}$) values for individual i and n_i_ is the subject-specific number of locations. The concordance criterion is especially suited for nonlinear models; it measures the level of agreement between the observed and predicted values and is a combined measure of the degree of accuracy and precision of pairs of values of y_i_ and on the identity line (the 45 degree line through the origin) and any CC ≤ 0 indicates lack of fit [35]

The strength of the Net Squared Displacement is that it provides an objective and quantitative basis for classifying different movement modes, as well as estimate movement characteristics such as extent, timing and duration of movements [24,26]. What is usually not tested in the literature is the sensitivity of such an approach to data characteristics and availability. For example movement data can often be incomplete with missing locations due to receiver problems, when animals are out of the transmission range, or collar malfunctions [27]. Missing locations might affect model-parameter estimates (example migration timing, duration, and extent), especially when missing locations fall in the critical change phases or animals are required to spend a considerable time at surface for allowing transmission [28–30]. Movement data is collected using devices such as GPS transmitters, geolocators, patagial GPS wing tags, pit tags, and many more. The data is recorded at intervals set *a priori* to correspond to a specific biological question. For instance, to study annual movement patterns of animals such as migration or a once-in-a-lifetime event (e.g. natal dispersal), sampling hourly locations may not add more value to results in comparison to sampling daily locations [6,27,31,32].

In most movement analyses, including migration, besides the extent and resolution issues mentioned above, one is encountered with the issue of selecting a start and end location for each tracked individual. This is primarily for defining the spatio-temporal scale of analyses such as migration patterns, seasonal home range use, daily or monthly activity patterns. The NSD is calculated from the first location and any errors in the starting location may result in the misclassification of movements and incorrect estimates for timing or distance. However, the effect of starting location on parameter estimates is rarely tested. The first starting location that the analyst deems appropriate, may have an effect either through its timing on, for example whether a species is in its winter range, summer range or on its migration path; or through its placement within an animal’s home range, i.e., centre of the range, towards the extremities, or even exploratory movements outside the home range. The first effect of a starting date may affect the model estimates if for example the starting date chosen for analysis is in the middle of a migratory period, especially when migratory phases are short. If this is the case, then the timing, extent and duration of migration may be incorrectly estimated. The second effect of starting location is determined by the method used to identify the first location in space, for example what is the effect of taking a mean location for a certain period instead of the actual first location of the animal, on estimates of migration distance, timing and duration? Certainly such subsampling may affect within-home range movements, or excursion movements, within seasonal ranges, or the estimates of distance moved. Although crucial for movement analyses, these questions have received little attention in the literature.

We designed this study to test the efficacy of the NSD approach in classifying different movement modes especially focusing on migration, along with three specific issues: A) *What is the effect of extent of movement of the species on it being classified as migrant*? We predict that the reliability of the model classification will be sensitive to the scale of movement and the individuals that move over large distance will be more reliably classified as migrants as opposed to short distance movers. B) *What is the effect of selected starting date and starting location of the animal on the movement mode classification and parameters*? If the selected starting date falls in the period when an animal has already started to migrate, then the model fits will be poor and the estimated migration characteristics of distance, timing and duration will be dubious. If the selected starting location falls outside of the animal’s normal home range, for example during exploratory movements, then the model fits and estimated migration characteristics will be less accurate than a mean starting location for a certain time period. C) *What is the effect of data resolution on the model fit and resulting interpretations*? For modeling seasonal migrations, a mean location per day may provide improved model fits compared with using a random location or multiple locations per day. A random location per day does not include all available movements during that time period, which may lead to inaccurate estimates of migration timing. In contrast, if individuals undertake short distance migrations, using multiple locations per day may complicate the model fitting process, as it becomes challenging to distinguish daily movements from migratory movements. Our intention is not to present an extensive comparison of different methods of analyzing movement data, but to show how study design aspects, as well as incorrect prior expectations, can produce dubious estimates from a popular method of quantifying migration. Similar caveats should also apply to the other methods of modeling migration.

## Materials and Methods

### Movement Data

The species used in this study is the moose (*Alces alces*) occurring in Sweden. The dataset available through WRAM (www.slu.se/wram) comprises 319 Global Positioning System (GPS) marked moose that were tracked at five study sites across a latitudinal gradient of 1500km (S1 Fig). The time period of tracking ranges from 01 March 2004 to 28 Febraury 2013, with a minimum of one location and maximum of 48 locations per day. Individuals that were tracked across multiple years had their trajectories split into years. The total number of single year trajectories available for this study was 489. Each moose year began in March, when most moose are in their winter range and ended the next March, or alternatively from September to September, when moose are in their summer range. The 41 individuals used in the second part of the study were all from the northern study sites shown in S1 Fig. The time period of tracking for these individuals was between 01 March 2008 and 28 February 2010.

### Methodological Framework

Our analysis has been divided into two parts in order to address the three questions: A) *What is the effect of extent of movement of the species on it being classified as migrant*? B) *What is the effect of starting date and starting location of the animal on the movement mode classification and parameters*? C) *What is the effect of data resolution on the model fit and resulting interpretations*?.The first part of the analyses addresses question A. This question is addressed by using a large dataset comprising 319 moose individuals from north of Sweden (63°N to 67°N). These individuals occur across a large latitudinal gradient of nearly 1500 km, thus experiencing varying environmental and climatic conditions. Accordingly, these individuals exhibit multiple movement modes, such as migration, nomadism, dispersal and being sedentary, while also varying in the extent, timing and duration of movements, providing an opportunity for assessing the performance of the NSD approach.

The second part of our analysis addresses questions B and C. A sample of the original dataset was used in this part of the study, consisting of 41 migratory individuals. These 41 individuals were tracked over the same time period continuously for a minimum of 2 years, at a resolution of 48 locations per day. The reasons for using a sub-sampled dataset include: (i) the issues raised in B and C above relate to migratory movements; (ii) to accurately test the effect of start date, a minimum period of two years of tracking was needed so that varying start dates could be used whilst maintaining a complete year of movements; (iii) large datasets can be computationally challenging for people running normal computers (example—practitioners), particularly when using high resolution data (48 locations per day).

### Classification of movements and characteristics

As a dependent variable, NSD estimates are fitted to movement models that can be used to describe the movement modes of migration, mixed migration, dispersal, nomadism and range residency (Table 1; [23]). In this context, migration is when an individual returns to the original location, whereas mixed migration is when an individual does not return to the original location but elsewhere (Table 1; [23]). Thus far, we have only referred to the commonly used metric of NSD (Net Squared Displacement). However, Börger & Fryxell [25] use Mean Squared Displacement (MSD) instead. MSD is the mean of the squared distance at each step over a given time period. Recent studies have not explored the differences as well as the potential implications of using the NSD or MSD. MSD provides smoother trajectories compared to NSD, which is highly variable due to daily movements within a territory or home range (S2 Fig). Consequently, fitting movement models to the MSD of a trajectory is simpler as annual movements can be more readily distinguished from daily movements, thus improving model convergence. However, MSD is inappropriate for estimating the model parameters of distance, timing and duration. Parameters derived from MSD are likely to reflect delayed timing of movements and increased durations (S2 Fig). Therefore, MSD may be used to initially classify different movement modes and once all movements have been classified, the models can be refitted to the NSD of the migratory individuals, such as migrants, to obtain distance, timing and duration of movements.

The models of dispersal, migration, mixed migration, remaining sedentary and nomadism were fitted to all data using nonlinear mixed-effect modeling (with MSD as the response variable), which allows for inclusion of variation between individuals for all model parameters–we used the nlme package with REML settings [33] within the R environment for statistical computing. The NSD of a trajectory was estimated using the AdehabitatLT library in R [34], and the MSD was estimated by taking a moving average of NSD from the previous 30 time steps. The number of time steps chosen will have an impact on the level of smoothing of the NSD of a trajectory. A small number of time steps will not provide much smoothing compared to the NSD whereas a high number of time steps may over-smooth the data meaning that migratory movements may not be detected. Migratory moose occur in their summer and winter home ranges for approximately 4 months (120 days). A window of 30 time steps did not risk a moose leaving its seasonal range before the moving average (MSD) had “caught up”. Model fit for each individual was evaluated using the concordance criterion (Table 1; [24,35]). The concordance criterion (CC) measures the level of agreement between the observed and predicted values, and is a combined measure of the degree of accuracy and precision of pairs of values of *y*_*i*_ and *ŷ*_*i*_ on the identity line (the concordance line or the 45° line through the origin; Table 1; [35]). CC ranges from -1 to 1, where CC values <0 indicate lack of fit and higher CC values indicate improved fit. The R code used for our analysis is provided in S1 File.

#### A) Extent of Movement

Results of the movement classifications were used to explore the effect that the extent of movement of a species has on model performance. We determined the extent of movement (NSD) and the accuracy of the model fit as determined by the CC value. We also determined whether movements had been classified correctly by visually inspecting the data [36]. Following this, we explored the relationship between the CC values (model fits) and MSD to determine whether model fit was sensitive to the extent of movement of individuals, which provided a good proxy of how extent of movements may influence movement classification.

#### B) Starting date and location effect

We selected 41 migratory individuals identified in the first part of the study, with a requirement that each individual had at least two years of continuous data. These two years of data were divided into twelve annual movement trajectories (i.e. containing one year of movements), such that each trajectory started in a different month and on the 1st of the month. Therefore, the first movement trajectory was from 01 March 2008 to 28 February 2009 and the final trajectory was from 01 February 2009 to 31 January 2010. We refitted the migratory movement model to these trajectories using NSD instead of MSD. The model outputs were used to calculate the spring and autumn migration dates (start and end of migration) as:

Migration Start=S+(θ−2φ)

Migration End=S+(θ+2φ)

where S is the starting date for the data, θ is the date that the spring/autumn migration reaches half its asymptotic height and φ is the timing (in days) elapsed between reaching ½ and ~¾ of the asymptote of the spring/autumn migration (Table 1). Börger & Fryxell [25] use 3φ to estimate the start and end point of movement phases. However, as φ is the timing elapsed between reaching ½ and ~¾, a value of 3φ may underestimate the start date and overestimate the end date of a migration (Table 1). Hence, we use 2φ which has also been used in a previous study to analyse the movements of a migratory species [36].

The migratory movement model consists of two legs/journeys, a spring and an autumn migration. Plotting the NSD of an annual migration path illustrates these two journeys (Fig 1), with a departure from the starting location, a stationary period and then a return to the starting location. The departure from the starting location is the first leg, or Migration 1, and this can either be spring or autumn, depending upon the starting date (as illustrated in Fig 1). Our study moves though a time series of multiple start dates; therefore we distinguish whether the seasonal migration estimates are generated from the first leg (Migration 1) or the second leg (Migration 2) of the NSD model.

The second component of the analysis was to explore the effect that ‘starting location’ has on the outcome of model estimates We investigate the effect of using a single starting location or a mean starting location. We used the same 41 individuals, but only used two of the annual datasets from above, namely movements starting in March’08 and September’08. We selected movements starting in March’08 and September’08 because around these dates moose are in their winter and summer home ranges respectively. Comparing these two time periods would allow us to explore whether the effect of a random or mean starting location varies between winter and summer movements of moose. In these datasets, the starting location for each moose was a single GPS location recorded close to 12:00 PM on the 1^st^ of the month, therefore, in essence, a random location within its home range or on its movement trajectory. Alternative starting locations were calculated for each moose by estimating its mean location during the first week and the first month. The mean starting locations were substituted into the NSD models and the results compared to determine how the starting location influenced the model predictions.

#### C) Data sampling / Resolution

The final component of the study was to understand how data resolution impacts the performance of NSD models and resulting interpretations. The resolution of data may vary from multiple locations per day, a single location per day or less than one location per day. High resolution data may overcomplicate the model fitting process whereas low resolution data may miss vital movement events, such as the initiation of migratory movements. To understand these effects, we apply three different levels of data resolution to the model fitting process. The March’08 and September’08 datasets were used again for this component of the study and the data resolutions used were: i) 1 random location per day; ii) between 6 and 48 locations per day (hereon high resolution); and iii) a mean location per day (hereon mean resolution), which is estimated by taking the harmonic mean of all locations in a day (i.e. the mean location of the high resolution data; see S3 Fig for an illustration of these three resolutions). We again used the NSD modelling approach to estimate the effect of data resolution on model estimates.

## Results

### Classification of movements

Approximately 47% of movements were classified as migratory, 11% as mixed migratory, 2% as nomadic, 6% as dispersal and 34% as resident (N = 489). However, these results include several movement trajectories that were reclassified, following manual inspection of the data. It is not uncommon for a movement to be misclassified using the NSD approach (see [36]). Therefore some restrictions were needed following the movement-modelling process, such as the classification of mixed-migratory individuals. We reclassified individuals identified as mixed-migratory if the return migration was within a certain distance of the origin (such as a distance related to the size of a home range), as this was in fact a migration. Alternatively, if the return migration was away from the origin, then this was in fact dispersal or nomadism. In addition to issues arising from the mixed-migratory model, we also had to reclassify several movements that were misclassified due to the extent of movement, which is discussed in more detail below. These restrictions were implemented after the analysis because it is not possible to place bounds on non-linear mixed effect models.

### Extent of movement

**Fig 2:**
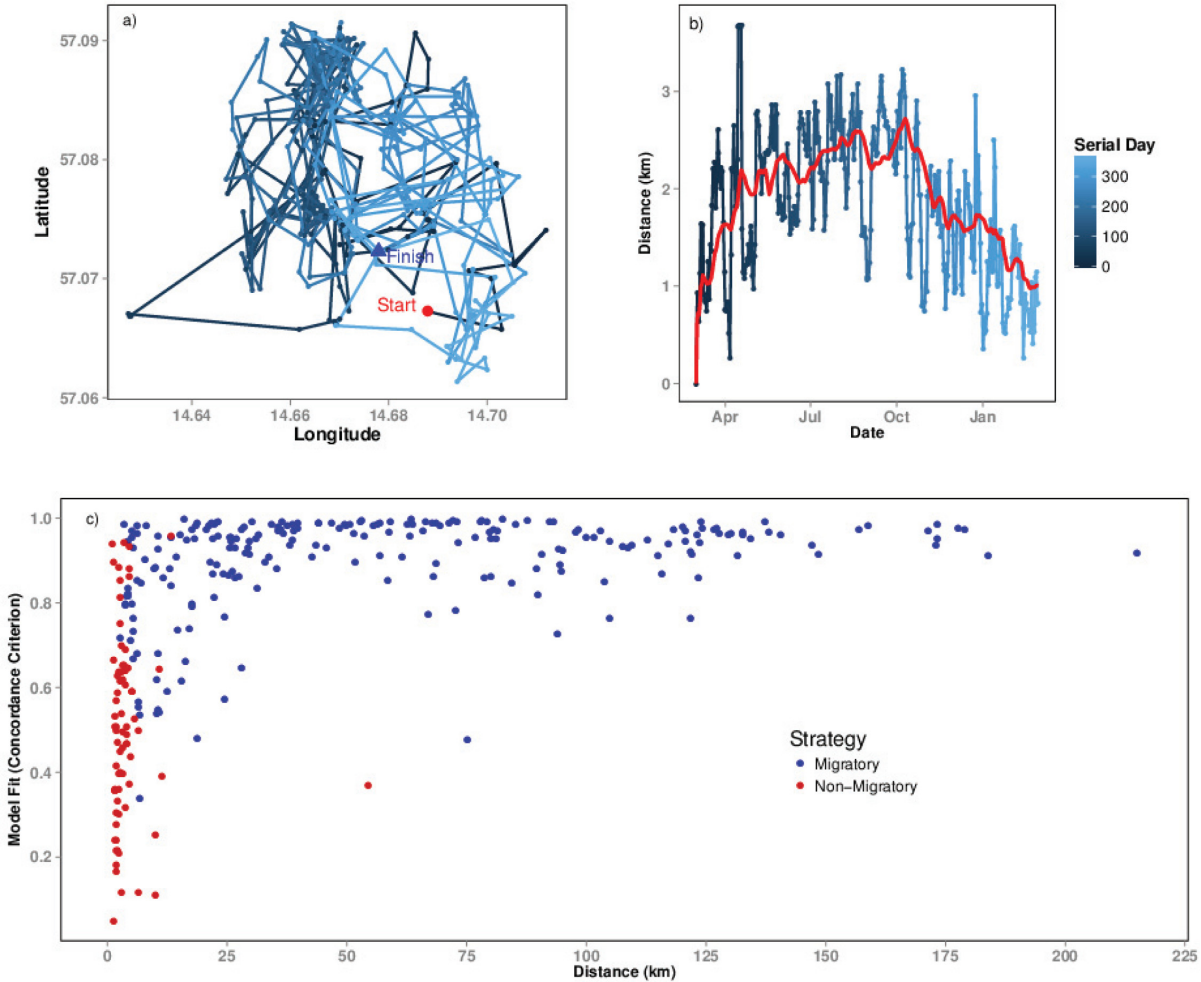
Classifying small-scale migrations. a) A movement trajectory and b) Net Squared Displacement (NSD) plot for an individual moose migrating a short distance. The coloured lines represent the movement over time, for both the xy path (a) and the NSD (b), whilst the red line indicates the MSD averaged over 30 time steps. It is evident from the deviations of the movement trajectory from the MSD, that for small-scale migrations, it is difficult to discriminate daily movements from migratory movements as these may operate over similar spatial scales. The MSD helps smooth the daily movements, thus improving the model fitting process, however, there is no easily identifiable summer range in comparison to Fig 1. c) Best Concordance Criterion (CC) value fitted against asymptote (scale of movement in kilometres) for moose classified as migratory (*n* = 299). Blue points indicate moose that were confirmed as migratory (*n* = 226) whereas red points indicate moose that had been misclassified as migratory (*n* = 73), as they were in fact non-migratory following visual inspection. The misclassified individuals had smaller scales of movement, and generally lower CC values. In addition, moose migrating at smaller scales (<10km) generally had poorer model fits in comparison to moose migrating at larger scales (>10km).

The extent of movement of the individual affected whether movement modes were classified correctly. This is particularly true for individuals migrating a distance that was similar to the distance of their daily movements (Fig 2). These movements complicate the model-fitting process, as it becomes a challenge to identify the extent of migration and to separate migratory movements from daily movements, particularly if individuals “visit” their former range (Fig 2). We also observed individuals being classified as migratory but were in fact resident. Reasons for this included increased movement during the rut, which produced migration-shaped NSDs, albeit very short migrations. Moreover, the timing of the return migration was occasionally greater than 365, thus falling outside the scope of the time period being analysed. Therefore these movements were in fact dispersal or home range movements. The incorrect classification of movements often occurred when no movement model would provide a good fit to the movement trajectory. This is illustrated in Fig 2, where the CC value of the NSD model improves as the annual net displacement increases. The majority of individuals that were misclassified as migratory, had extents of movement less than 10km and CC values that were less than 0.7 (mean = 0.50, sd = 0.23). In contrast the average CC for migratory individuals is much higher (mean = 0.89, sd = 0.12), with the CC value increasing as the extent of movement increases.

### Starting Date

**Fig 3:**
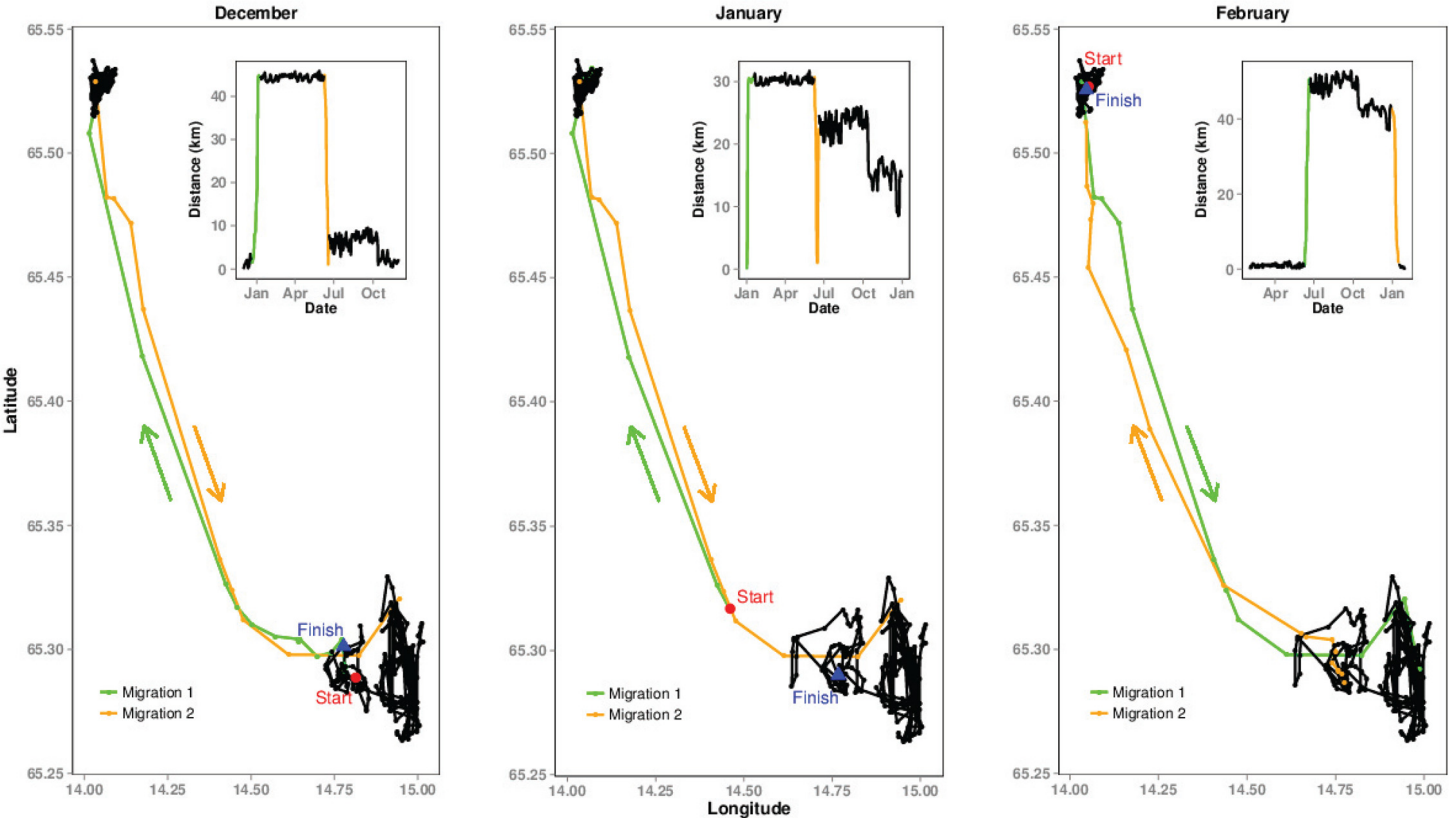
The effect of start date on movement classification. Movement trajectories and Net Squared Displacement plots of a moose individual showing the effect of start date (December, January and February). In December, the individual is still in the summer range providing a standard NSD for migration; in January, the moose had already begun its migration with subsequent impacts on the shape of the NSD; in February, the moose was in its winter range after having completed its migration, once again providing a standard NSD for migration.

The results described from hereon are only for the 41 migratory individuals identified in the first part of the analysis. The average migration distance for these individuals was 64 km (SD = 52km), with a range from 3 km to 172 km, thus providing variable extents of movement for the second part of the analysis. After refitting the migratory movement model using NSD, we identified the timing of migrations using the model results for the 12 datasets of March’08 through to February’09 (Table 2). These results move through a series of spring and autumn migrations as can be seen in the migration ‘from’ and ‘to’ dates. Migration predictions appear to be robust to starting date apart from a few exceptions, namely the datasets beginning in June, December and January. In the study region, the majority of migrations appear to begin in December–January or June (Table 2). Therefore, using starting values during this period will provide a sample of moose that are mixed between those in their winter range, summer range or on their migratory journey. This is clearly illustrated in Fig 3, by comparing the NSD plots of December, January and February and also confirmed in Table 3, where 36% of moose had not yet completed their spring migration by the start of June. Based on these results, the optimal start date to select, for identifying the onset of spring migrations, is when moose are in their winter range (i.e. February to May) and for autumn migrations, is when they are in their summer range (August to November).

**Table 2:** The impact of “Starting Date” on the model outputs of migration parameters. Results are the averaged output for the 41 moose used in this study. The model outputs show estimates of distance (km^2^), timing (“From” is the start date of migration and “To” is the end date of migration) and duration (number of days) of the migrations. As per Fig 1, Migration 1 or 2 can be either a spring or autumn migration depending on the starting date of the data. For moose in northern Sweden, spring migrations occur in May and June and autumn migrations occur between November and January. For the quantification of start and end of migration, we used the formulas mentioned in Table 1.

		Migration 1			Migration 2		
Dataset	Distance (km^2^)	From	To	Duration	From	To	Duration
**Mar'08**	9892	02/06/2008	20/06/2008	18	10/11/2008	18/12/2008	39
**Apr'08**	9616	30/05/2008	17/06/2008	18	07/11/2008	19/12/2008	42
**May'08**	10003	02/06/2008	21/06/2008	19	06/11/2008	18/12/2008	42
**Jun'08**	6158	05/10/2008	26/10/2008	21	16/03/2009	12/04/2009	27
**Jul'08**	8237	01/12/2008	24/12/2008	23	22/04/2009	13/05/2009	21
**Aug'08**	10301	02/12/2008	27/12/2008	24	29/04/2009	31/05/2009	31
**Sep'08**	9485	02/12/2008	28/12/2008	26	06/05/2009	26/05/2009	20
**Oct'08**	9528	12/12/2008	05/01/2009	24	08/05/2009	31/05/2009	23
**Nov'08**	7873	14/12/2008	09/01/2009	26	08/05/2009	01/06/2009	24
**Dec'08**	8554	16/01/2009	13/02/2009	28	18/06/2009	23/07/2009	35
**Jan'09**	7712	01/04/2009	20/04/2009	18	10/09/2009	22/11/2009	73
**Feb'09**	10461	25/05/2009	11/06/2009	17	10/11/2009	09/01/2010	61

**Table 3:** Effect of selection of “Starting Date” for different months and the predictability of migration. The table shows the percentage of moose individuals (*n* = 26) that are in their winter or summer range.

**Range**	**Jan**	**Feb**	**Mar**	**Apr**	**May**	**Jun**
**Winter**	68	100	100	100	100	36
**Summer**	32	0	0	0	0	64
**Range**	**Jul**	**Aug**	**Sep**	**Oct**	**Nov**	**Dec**
**Winter**	4	0	0	0	0	24
**Summer**	96	100	100	100	100	76

### Starting Location

For the March’08 dataset, changing the first data point from a single location to a harmonic mean location resulted in minor differences in the NSD model outputs, for both mean location during the first week (SLW) and during the first month (SLM; Table 4). There was a small improvement in the model fit (CC values) and minimal variation in the distance (asymptote) and timing of migrations. The September’08 dataset showed greater variation in model outputs when changing the first data point from a single location to the harmonic mean location (Table 4). Again, there were minor improvements in model fit (CC values) but unlike the March’08 dataset, there was some variation in both the distance (asymptote) and timing of migrations. The distance decreases and the timings of Migration 1 are delayed, becoming more closely aligned to the Oct’08 and Nov’08 predictions in Table 2.

**Table 4:** Comparison of migration model outputs at differing temporal resolutions of movement data and spatial resolution of starting locations using first recorded location and single location per day (SL), single location per day but a starting location that is the mean location during the first week (SLW), single location per day but a starting location that is the mean location during the first month (SLM) mean resolution data (MR) or high resolution data (HR).

Dataset	Extent	Mig1 St	Mig1 End	Mig2 St	Mig2 End	Model CC
Mar08 SL	9,893	02/06/2008	20/06/2008	10/11/2008	18/12/2008	0.962
Mar08 SLW	9,879	01/06/2008	19/06/2008	10/11/2008	19/12/2008	0.965
Mar08 SLM	9,898	02/06/2008	20/06/2008	10/11/2008	19/12/2008	0.964
Mar08 MR	9,614	31/05/2008	17/06/2008	12/11/2008	15/12/2008	0.964
Mar08 HR	9,895	26/05/2008	21/06/2008	15/11/2008	19/12/2008	0.940
Sep08 SL	9,486	02/12/2008	28/12/2008	06/05/2009	26/05/2009	0.974
Sep08 SLW	9,339	06/12/2008	26/12/2008	06/05/2009	27/05/2009	0.975
Sep08 SLM	8,797	10/12/2008	29/12/2008	07/05/2009	26/05/2009	0.980
Sep08 MR	9,695	08/12/2008	30/12/2008	07/05/2009	27/05/2009	0.975
Sep08 HR	9,463	30/11/2008	01/01/2009	08/05/2009	05/06/2009	0.966

**Fig 4:**
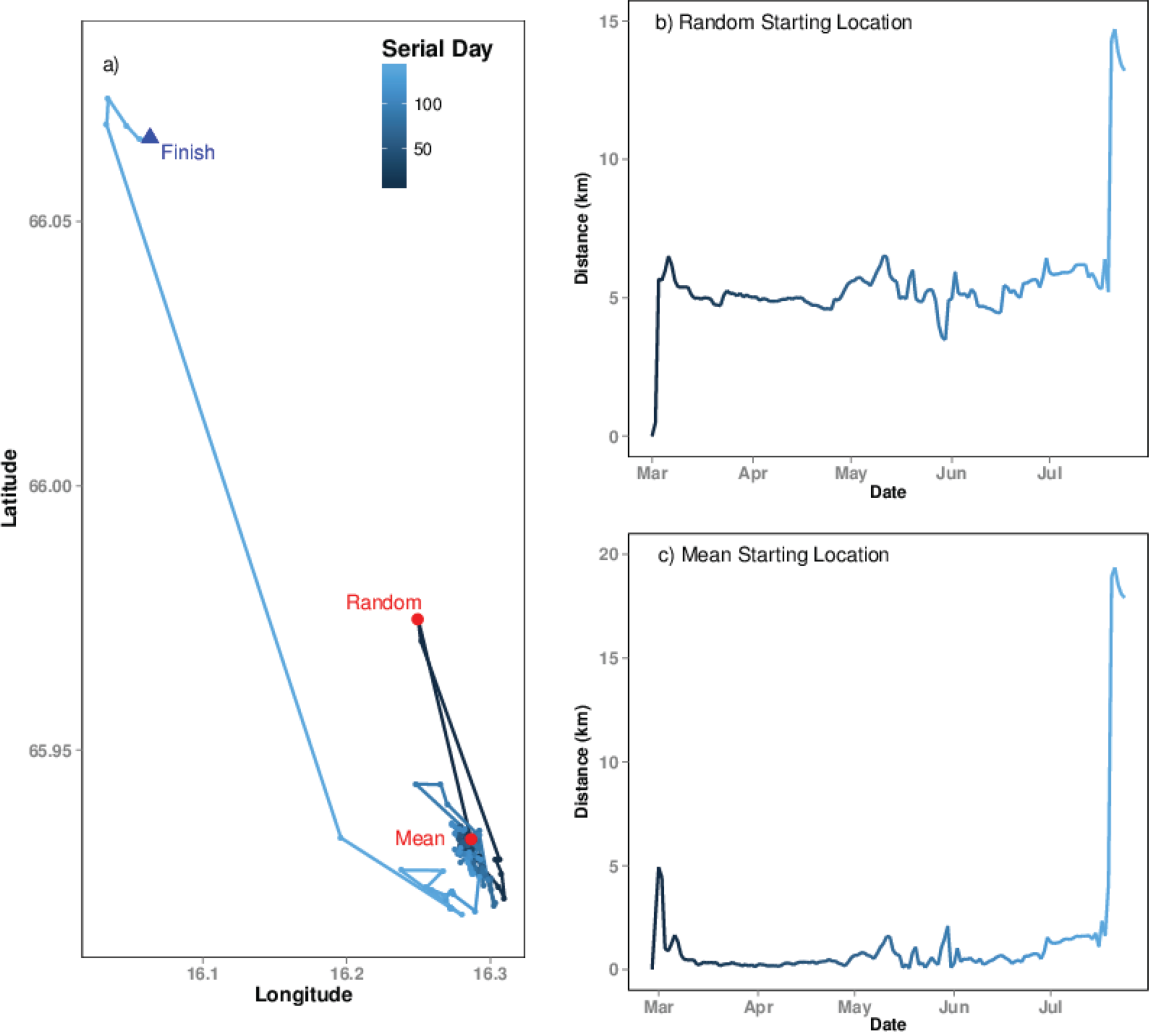
The effect of using a random starting location or a mean starting location. a) shows the XY path of the first few months of a movement trajectory for a moose individual, the random starting location and mean starting location are shown in red. The effects can also be seen in the Net Squared Displacement of the individual if the random starting location (b) is compared to the mean starting location (c). It should be noted that the random starting location was an actual location used by the animal and implies how this may identify locations that fall outside home range.

**Fig 5:**
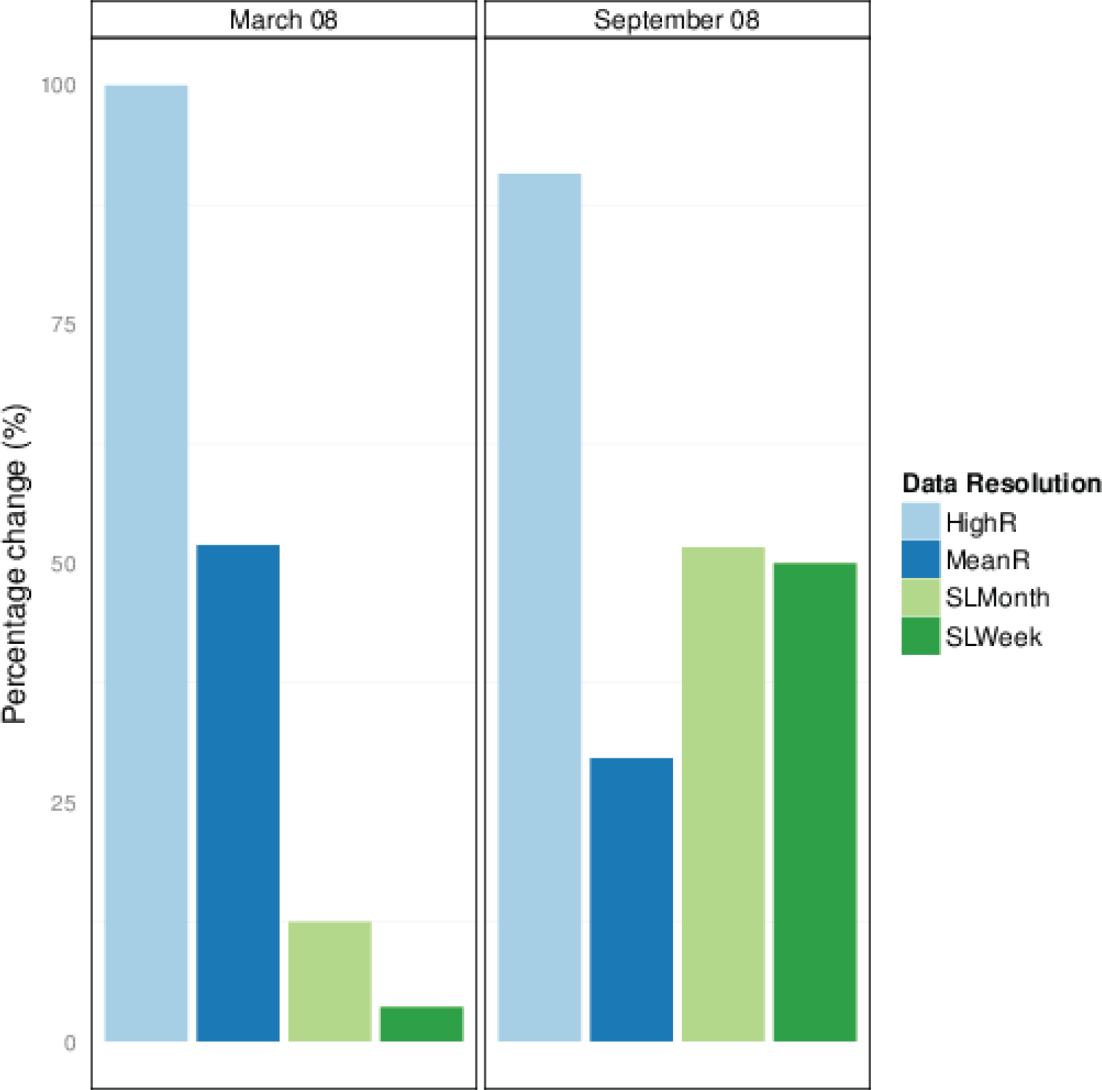
The effects of data resolution on model estimates. Percentage of migration timings that changed by more than 1 day in comparison to estimates using a single location per day with a random starting location. Starting dates were in March’08 and September’08 using data resolutions of high-resolution (HighR), mean-resolution (MeanR), single location per day with a mean starting location during the first month (SLMonth) or single location per day with a mean starting location during the first week (SLWeek). High-resolution data altered the timing of migrations for most individuals by more than 1 day, whereas using mean-resolution data altered the timing of migrations by more than 1 day for 50% of individuals with a starting date in March’08 and approximately 30% in September’08. Using a mean starting location had a negligible effect on the timing of migrations when the start dates were in March’08, but there was a greater influence on timing estimates for start dates in September’08.

At the individual level, using a mean starting location as the first data point may result in an altered NSD curve, such as the individual shown in Fig 4. This effect may influence the model estimates for distance and timing of migrations. A mean starting location during the first month generally resulted in greater variation in model outputs than a mean location during the first week (Fig 5A). Using a mean starting location during the first week or month, influenced the analysis of the Sep’08 dataset much more, where approximately 25% to 40% of moose had an earlier or delayed Migration 1 by >3 days (Fig 5A), and the most extreme was by 43 days. Using a mean starting location, for both week and month, influenced the analysis of the March’08 dataset to a lesser degree, although one individual’s timing was delayed by 34 days (Fig 4). However, for both the Mar’08 and Sep’08 datasets, the timing for the autumn migrations had the most variation (Autumn migrations are Migration 2 in Mar’08 and Migration 1 in Sep’08).

### Resolution

Model fits were worse (<CC) when using the high-resolution data compared to one location per day and the model that included *distance*, *timing* and *duration* parameters (Table 1) as random effects did not converge due to large variation between individuals (Table 4). Hence, the best model did not include *duration* parameter as a random effect. The model fits using a mean resolution were very similar to the models using one location per day (CC values, Table 4). In addition, in this case it was also possible to fit a model with *distance*, *timing* and *duration* parameters as random-effects (Table 1). The most important effect of using the high-resolution data was on the timing. As it was not possible to include the *duration* parameter in the random effects model structure, the migration estimates for the high resolution data have substantial variation from those using one location per day, with the timing estimates varying by >3 days for over 75% of moose (Fig 5B). There is less variation in model outputs when using a mean resolution, however 50% of moose had timings that varied between 1 and 3 days in the March dataset, and in almost all of these, the start and end dates were approximately 1 day earlier for Migration 1.

## Discussion

We show that the results of movement analyses using NSD are sensitive to the extent of movement of animals, starting date, starting location and the temporal resolution of the data. For correctly classifying migratory movements, the modelling approach evaluated here works much more efficiently for longer distance migrants and the use of MSD was more effective inferentially and statistically, than NSD. This is because the MSD produces smoother curves that are easier to fit in contrast to NSD curves, which have sharp drops and rises and are therefore harder to fit using the smoother non-linear functions. Moreover, since the aim in this step is to be able to distinguish between the shapes of the curve rather than quantify the curve parameters, it is inferentially more advantageous. In the case of moose, which is a partially migratory species, the models performed better for animals that travelled >10 km. For animals that moved <10km, model predictions were mixed and more variable, hence warranting careful inspection of trajectories. Such a finding suggests the need for discussion about how migration is defined and then adapt statistical models accordingly. Studies like [16] considered animals as ‘migrants’ whose seasonal ranges were separated by >10km. Our results provide strong evidence that such arbitrary cut-offs may not be adopted when species, populations or individuals show such large variations in extents of movements [23,24].

Similarly, Mysterud et al. [36] modelled movements of red deer (*Cervus elaphus*) using the NSD approach, a species with similar characteristics to the moose in terms of being partially migratory with variable extents of movement and migratory distance. Mysterud et al. [36] found that the NSD method misclassified movement modes, even for those individuals with non-overlapping seasonal ranges. They also reported errors during the estimation of migration parameters for individuals designated as migratory, due to the timing of migration being misclassified by the NSD model. Clearly, we demonstrate here, how the extent of movement can affect the movement parameters obtained from NSD approach and fitting models with MSD may be better at least to distinguish between the movement modes.

Our analysis of starting dates indicates that the models were robust to starting values, provided the animal was in either its winter or summer home range and not when the animal has most probably started migrating (January, June and December). Therefore, the optimal start dates for a species such as moose, which are mostly captured in winter or early spring, would be when an animal is in its summer range (i.e. July to October for this dataset) or winter range (February to April for this dataset). It is understandable that the modelling method, which uses non-linear mixed effects models, will struggle to converge if the starting data points are in the middle of the slope, i.e. during the migratory phase of the individual.

The movement models struggled to converge when moose were in their summer ranges (i.e. a start date between August and November) and also showed large variability in onset of migration predictions. Different start dates during this period may give highly variable results due to many reasons. Terrestrial migratory species may demonstrate more flexible movement strategies in autumn compared to spring, especially in relation to food availability and snow conditions, where they may maximize energy intake rather than minimizing time, and hence delay autumn migration until the conditions in the summer range become unfavourable. Sawyer et al. [37] report similar findings for migratory mule deer and we observe the same in moose. Conditions in wintering areas and stop-over sites may, however, also influence an individual’s decision to migrate [38,39]. In addition, disturbance during hunting, loss of offspring, as well as increased activity of moose, may also explain such high variability in the onset of individuals’ migration dates [24,40]. Such erratic movements may be problematic when fitting statistical models, which work under certain assumptions. For example, the NSD migration model assumes a return to the original starting point. If an individual returns to a point that is farther away, even if this is within the seasonal home range, the NSD will not return to zero and these points on the tail of the model will increase the *duration* parameter (Table 1), and therefore the predicted migration dates. This is evident in the long duration (mean 40 days) observed in autumn migration dates for the February to May datasets compared to the July to November datasets (mean 24 days, contrast the Migration 2 results for Feb’08 to May’08 with Migration 1 results for Sep’08 to Nov’08 in Table 2, all these predictions are for the “autumn” migration in 2008). Where individuals do not return to the origin, the mixed migratory model may be more suitable as it contains an additional parameter to allow for individuals returning to a different location (Table 1; [23]).

Using a mean starting location improves model performance compared to using a single location. As shown through the example in Fig 4, it is possible that the first point captured in a trajectory is an exploratory movement rather than within the home range, which is better represented by the mean starting location. The choice of a mean starting location during the first week or month appears to interact with date, a mean starting location during the first week improved the model output when using a Mar’08 or Spring start date, as moose generally move less during winter. However, a mean starting location during the first month improved the model output in Sep’08 dataset and resulted in changes in migration predictions. The mean location identifies the centre of the home range and may include an area that is not used by the target species. NSD models distance over time, thus we do not feel it is essential for the starting location to be a used site since the model is not estimating resource use. However, an alternative to a mean location per day is the median geographical location, which estimates the approximate centre of the home range and does include a site used by the species. One must also consider the biology of a species. For example, using a mean location may be less appropriate for species with clear roosting sites or colonies, such as bats or seals. The mean location should also be removed from subsequent analyses once the movement strategy of the individual has been identified. Using a mean starting location may nullify the erratic September movements (in case of moose), which could be better for the model, but then one must be careful about the species in question and its biology.

For modeling annual migrations, one location per day (selected based on a time criteria, such as time of the day) or mean location per day (geographic mean of all locations in a day) appeared to be more appropriate than high resolution data, especially when modeling movements of animals that migrate shorter distances. High-resolution data did not improve on model outcomes for modelling migration. In fact, high-resolution data worsened the predictions due to the simpler model that does not account for individual variation in the distance, timing and duration of migration. Instead, mean resolution data were more computationally tractable; they incorporated all available data and permitted use of a smaller sample size of a single location per day. The mean location may not include a site used by the species, which should not be an issue for estimating annual movement patterns, but an alternative would be to use the geographic median location to include a used site. In marine environments, tracking data is often sparse, restricting the number of locations to a few per day or a single location a day for each individual. Our approach can certainly be useful for marine animal migration studies [5]. Nevertheless, preparation time is needed to compute the mean resolution, and as has been shown, there is not much variation in model outputs when this is done, so is it necessary? Migration is an annual event and studies addressing migration characteristics should ideally select locations as per the question. Nams [41] suggests that animals perceive and react to the habitat heterogeneity and structure at many different spatial scales (extents as we refer to in this paper) and accordingly, their biology (e.g. foraging behaviour, dispersal patterns, orientation, and population dynamics relate to the sections of these scales). Nams [41] referred to such sections as ‘‘domains”, and that heterogeneity can exist at spatial scale. For example, an animal may travel towards a certain place and forage for food on the way. There would be two domains: at the large scale the animal travels, and on the small scale the animal forages. In the large domain the movement pattern is homogeneous, as the animal travels in a directed walk; in the small domain the movement pattern is heterogeneous, as the animal enters and leaves patches of food. Hence, as per Nams [41], low(er) resolution data would capture migration, which would fall in the larger domain where the animal travels whereas high resolution data captures both the small and large scale domains where the animal forages and travels.

A final consideration is how GPS location error may potentially effect inferences made from the NSD approach. GPS location error may reduce our ability to detect behavioural patterns, such as migratory behaviours [42]. A number of factors may influence the precision of location estimates like vegetation cover or topography for terrestrial species [43] and time spent at the surface for marine species [29]. The effect of location error will depend upon the scale of movement of the species [42]; a location error of 30m will have little impact on NSD estimates of species migrating over 100km. However, location error may be much larger for marine species and from other tracking devices like light based geolocators [29,44]. The large location errors from these tracking devices may reduce model convergence whilst using the NSD approach, and may result in misclassified movements and erroneous parameter estimates. Some of the approaches discussed in this study may reduce the effect of GPS location error, such as using the MSD to smooth trajectories, using a mean starting location or using a mean or median location per day. A number of modelling approaches have also been developed to remove GPS location errors and improve GPS accuracy, thus reducing the effect of GPS location errors [45,46].

We hereby provide guidance on designing, analysing and interpreting migration ecology studies, especially those that aim to use the NSD approach. We suggest that arbitrary cut-offs to distinguish seasonal and migratory ranges may not be adopted when individuals within a species shows large variations in extents of movements; the extent of movement can affect the movement parameters obtained from NSD approach. We recommend using MSD to classify movement trajectories as migratory, sedentary, dispersal or nomadism. MSD may also be useful in studies with larger GPS location errors as model convergence is improved and GPS error is smoothed. The level of smoothing for the MSD, i.e. the number of time-steps used for the moving average, should provide a smoother trajectory without compromising the detection of seasonal home ranges. MSD is inappropriate for estimating model parameters of distance, timing and duration and NSD should be used for this step. One should ensure that the starting date used for analysis is not during a time period when animals are migrating, but instead when the animal is in its seasonal range. In the case of moose, it was important to use a mean starting location when moose are in their summer range due to increased movements during this time whereas this was not necessary if a winter starting location was used. However, a mean starting location during winter would reduce the possibilities of selecting a location that was outside an individual’s normal range of movements. A single location or mean location per day may be enough to identify migrants and estimate migration parameters using the NSD approach. This result may be important for studies in other taxa, such as birds or marine species, which may only have a few locations per day.

The choice of data collection, analyses and interpretation of results should be determined by the biological question, focal species and its life history and scale of movements. Fryxell et al. [47] demonstrate a nice example of selecting the scale of data to match the biological question and scale of movements. However, many studies still do not necessarily reveal the rationale behind selecting a particular number of locations for their analyses [14,48]. There is an urgent need to answer basic questions on movement characteristics and how they extend from individuals to population and ecosystem-level consequences [49]. Moreover, for managers, these estimates are critical to designing protection efforts and allocating resources, and especially in identifying the optimal scale of management [8,50,51].

## Supporting Information

S1 Fig Study Map. The locations of the moose individuals included in this study overlaid on the map of Sweden.
(EPS)

S2 Fig MSD versus NSD. A comparison of a movement trajectory shown with the net-squared displacement (NSD; shaded blue) and the mean-squared displacement (MSD; shaded red). The MSD was calculated using a moving average of 14 days.
(EPS)

S3 Fig Illustration of data resolution. A comparison of a movement trajectory shown at high resolution (left; 1 location every hour), 1 location per day (center) and a mean location per day (right). The mean location per day was estimated by taking the geographic mean of locations.
(EPS)

S1 File R code. The R code used for our analysis.
(R) Click here for additional data file.

S2 File Movement data for NSD models. Zipped folder containing four data files. Each file contains a unique ID for the individuals, a timestamp (nDaysYr) and the NSD which are necessary to fit the models outlined in S1 File. The data files contain 1) Movement data for part one (classifying 489 trajectories), 2) Movement data for part two with 1 location per day, 3) Movement data for part two with high resolution and 4) Movement data for part two with mean resolution. All data files for Part Two contain 41 individuals as outlined in the methods.
(ZIP)

## References

[1] Kays R, Crofoot MC, Jetz W, Wikelski M. Terrestrial animal tracking as an eye on life and planet. Science (80-). 2015;348: aaa2478 10.1126/science.aaa2478 26068858

[2] Hussey NE, Kessel ST, Aarestrup K, Cooke SJ, Cowley PD, Fisk AT, et al Aquatic animal telemetry: A panoramic window into the underwater world. Science. 2015;348: 1255642 10.1126/science.1255642 26068859

[3] Dettki H, Ericsson G. Screening Radiolocation Datasets for Movement Strategies With Time Series Segmentation. J Wildl Manage. 2008;72: 535–542. 10.2193/2006-363

[4] Cagnacci F, Boitani L, Powell RA, Boyce MS. Animal ecology meets GPS-based radiotelemetry: a perfect storm of opportunities and challenges. Philos Trans R Soc B Biol Sci. 2010;365: 2157–2162. 10.1098/rstb.2010.0107 PMC2894970 20566493

[5] Schofield G, Dimadi A, Fossette S, Katselidis KA, Koutsoubas D, Lilley MKS, et al Satellite tracking large numbers of individuals to infer population level dispersal and core areas for the protection of an endangered species. Divers Distrib. 2013;19: 834–844. 10.1111/ddi.12077

[6] Pendoley KL, Schofield G, Whittock PA, Ierodiaconou D, Hays GC. Protected species use of a coastal marine migratory corridor connecting marine protected areas. Mar Biol. 2014;161: 1455–1466. 10.1007/s00227-014-2433-7

[7] Hays GC, Mazaris AD, Schofield G. Different male vs. female breeding periodicity helps mitigate offspring sex ratio skews in sea turtles. Front Mar Sci. 2014;1: art43 10.3389/fmars.2014.00043

[8] Allen AM, Singh NJ. Linking Movement Ecology with Wildlife Management and Conservation. Front Ecol Evol. 2016;3: art155 10.3389/fevo.2015.00155

[9] Bolger DT, Newmark WD, Morrison TA, Doak DF. The need for integrative approaches to understand and conserve migratory ungulates. Ecol Lett. 2008;11: 63–77. 10.1111/j.1461-0248.2007.01109.x 17897327

[10] Holdo RM, Fryxell JM, Sinclair ARE, Dobson A, Holt RD. Predicted Impact of Barriers to Migration on the Serengeti Wildebeest Population. PLoS One. 2011;6: e16370 10.1371/journal.pone.0016370 21283536 PMC3026817

[11] Middleton AD, Kauffman MJ, Mcwhirter DE, Cook JG, Cook RC, Nelson AA, et al Animal migration amid shifting patterns of phenology and predation: Lessons from a Yellowstone elk herd. Ecology. 2013;94: 1245–1256. 10.1890/11-2298.1 23923485

[12] Chapman BB, Brönmark C, Nilsson J-Å, Hansson L-A. The ecology and evolution of partial migration. Oikos. 2011;120: 1764–1775. 10.1111/j.1600-0706.2011.20131.x

[13] Singh NJ, Leonardsson K. Partial Migration and Transient Coexistence of Migrants and Residents in Animal Populations. PLoS One. 2014;9: e94750 10.1371/journal.pone.0094750 24722396 PMC3983253

[14] Beatty WS, Kesler DC, Webb EB, Raedeke AH, Naylor LW, Humburg DD. Quantitative and Qualitative Approaches to Identifying Migration Chronology in a Continental Migrant. PLoS One. 2013;8: 1–9. 10.1371/journal.pone.0075673 PMC3794004 24130732

[15] Cagnacci F, Focardi S, Heurich M, Stache A, Hewison AJM, Morellet N, et al Partial migration in roe deer: migratory and resident tactics are end points of a behavioural gradient determined by ecological factors. Oikos. 2011;120: 1790–1802. 10.1111/j.1600-0706.2011.19441.x

[16] Hjeljord O. Dispersal and migration in northern forest deer-are there unifying concepts? Alces. 2001 pp. 353–370.

[17] Morales JM, Haydon DT, Frair J, Holsinger KE, Fryxell JM. Extracting more out of relocation data: building movement models as mixtures of random walks. Ecology. 2004;85: 2436–2445. 10.1890/03-0269

[18] Jonsen ID, Flemmings JM, Myers R a. Robust State–Space Modeling of Animal Movement Data. Ecology. 2005;86: 2874–2880. 10.1890/04-1852

[19] Jonsen ID, Basson M, Bestley S, Bravington M V., Patterson TA, Pedersen MW, et al State-space models for bio-loggers: A methodological road map. Deep Res II. Elsevier; 2013;88–89: 34–46. 10.1016/j.dsr2.2012.07.008

[20] Patterson T, Thomas L, Wilcox C, Ovaskainen O, Matthiopoulos J. State–space models of individual animal movement. Trends Ecol Evol. 2008;23: 87–94. 10.1016/j.tree.2007.10.009 18191283

[21] Fleming CH, Calabrese JM, Mueller T, Olson KA, Leimgruber P, Fagan WF. From Fine-Scale Foraging to Home Ranges: A Semivariance Approach to Identifying Movement Modes across Spatiotemporal Scales. Am Nat. 2014;183: E154–E167. 10.1086/675504 24739204

[22] Jonsen ID, Myers RA, James MC. Robust hierarchical state-space models reveal diel variation in travel rates of migrating leatherback turtles. J Anim Ecol. 2006;75: 1046–1057. 10.1111/j.1365-2656.2006.01129.x 16922840

[23] Bunnefeld N, Börger L, van Moorter B, Rolandsen CM, Dettki H, Solberg EJ, et al A model-driven approach to quantify migration patterns: individual, regional and yearly differences. J Anim Ecol. 2011;80: 466–476. 10.1111/j.1365-2656.2010.01776.x 21105872

[24] Singh NJ, Börger L, Dettki H, Bunnefeld N, Ericsson G, Borger L, et al From migration to nomadism: Movement variability in a northern ungulate across its latitudinal range. Ecol Appl. 2012;22: 2007–2020. 10.1890/12-0245.1 23210316

[25] Börger L, Fryxell JM. Quantifying individual difference in dispersal using net squared displacement In: Colbert J, Baguette M, Benton T, Bullock J, editors. Dispersal Ecology and Evolution. Oxford University Press; 2012 pp. 221–230.

[26] Allen AM, Månsson J, Jarnemo A, Bunnefeld N. The impacts of landscape structure on the winter movements and habitat selection of female red deer. Eur J Wildl Res. 2014;60: 411–421. 10.1007/s10344-014-0797-0

[27] Nielson RM, Manly BFJ, Mcdonald LL, Sawyer H, Mcdonald TL. Estimating habitat selection when GPS fix success is less than 100%. Ecology. 2009;90: 2956–2962. 10.1890/08-1562.1 19886504

[28] Gurarie E, Andrews R, Laidre KL. A novel method for identifying behavioural changes in animal movement data. Ecol Lett. 2009;12: 395–408. 10.1111/j.1461-0248.2009.01293.x 19379134

[29] Ryan PG, Petersen SL, Peters G, Gremillet D. GPS tracking a marine predator: the effects of precision, resolution and sampling rate on foraging tracks of African Penguins. Mar Biol. 2004;145: 215–223. 10.1007/s00227-004-1328-4

[30] Schofield G, Bishop CM, MacLean G, Brown P, Baker M, Katselidis KA, et al Novel GPS tracking of sea turtles as a tool for conservation management. J Exp Mar Bio Ecol. 2007;347: 58–68. 10.1016/j.jembe.2007.03.009

[31] Hebblewhite M, Haydon DT. Distinguishing technology from biology: a critical review of the use of GPS telemetry data in ecology. Philos Trans R Soc Lond B Biol Sci. 2010;365: 2303–2312. 10.1098/rstb.2010.0087 20566506 PMC2894965

[32] Maxwell SM, Hazen EL, Bograd SJ, Halpern BS, Breed G a, Nickel B, et al Cumulative human impacts on marine predators. Nat Commun. Nature Publishing Group; 2013;4: 2688 10.1038/ncomms3688 24162104

[33] Pinheiro J, Bates DM. Mixed Effect Models in S and S-PLUS. New York: Springer; 2000.

[34] Calenge C, Dray S, Royer-Carenzi M. The concept of animals’ trajectories from a data analysis perspective. Ecol Inform. Elsevier B.V.; 2009;4: 34–41. 10.1016/j.ecoinf.2008.10.002

[35] Huang S, Meng SX, Yang Y. Assessing the goodness of fit of forest models estimated by nonlinear mixed-model methods. Can J For Res. 2009;39: 2418–2436. 10.1139/X09-140

[36] Mysterud A, Loe LE, Zimmermann B, Bischof R, Veiberg V, Meisingset E. Partial migration in expanding red deer populations at northern latitudes—a role for density dependence? Oikos. 2011;120: 1817–1825. 10.1111/j.1600-0706.2011.19439.x

[37] Sawyer H, Kauffman MJ, Nielson RM, Horne JS. Identifying and prioritizing ungulate migration routes for landscape-level conservation. Ecol Appl. 2009;19: 2016–2025. 10.1890/08-2034.1 20014575

[38] Lendrum PE, Anderson CR, Monteith KL, Jenks JA, Bowyer RT. Migrating Mule Deer: Effects of Anthropogenically Altered Landscapes. PLoS One. 2013;8: e64548 10.1371/journal.pone.0064548 23691246 PMC3653929

[39] White KS, Barten NL, Crouse S, Crouse J. Benefits of migration in relation to nutritional condition and predation risk in a partially migratory moose population. Ecology. 2014;95: 225–237. 10.1890/13-0054.1 24649661

[40] Singh NJ, Ericsson G. Changing motivations during migration: linking movement speed to reproductive status in a migratory large mammal. Biol Lett. 2014;10: 1–4. 10.1098/rsbl.2014.0379 PMC4090558 24942710

[41] Nams VO. Using animal movement paths to measure response to spatial scale. Oecologia. 2005;143: 179–188. 10.1007/s00442-004-1804-z 15657759

[42] Bradshaw CJA, Sims DW, Hays GC. Measurement Error Causes Scale-Dependent Threshold Erosion of Biological Signals in Animal Movement Data. Ecol Appl. 2007;17: 628–638. 10.1890/06-0964 17489266

[43] Hansen MC, Riggs R a. Accuracy, precision, and observation rates of global positioning system telemetry collars. J Wildl Manage. 2008;72: 518–526. 10.2193/2006-493

[44] Lisovski S, Hewson CM, Klaassen RHG, Korner-Nievergelt F, Kristensen MW, Hahn S. Geolocation by light: accuracy and precision affected by environmental factors. Methods Ecol Evol. 2012;3: 603–612. 10.1111/j.2041-210X.2012.00185.x

[45] Bjørneraas K, Van Moorter B, Rolandsen CM, Herfindal I. Screening Global Positioning System Location Data for Errors Using Animal Movement Characteristics. J Wildl Manage. 2010;74: 1361–1366. 10.2193/2009-405

[46] Lowther AD, Lydersen C, Fedak MA, Lovell P, Kovacs KM. The Argos-CLS Kalman Filter: Error Structures and State-Space Modelling Relative to Fastloc GPS Data. PLoS One. 2015;10: e0124754 10.1371/journal.pone.0124754 25905640 PMC4408085

[47] Fryxell JM, Hazell M, Börger L, Dalziel BD, Haydon DT, Morales JM, et al Multiple movement modes by large herbivores at multiple spatiotemporal scales. Proc Natl Acad Sci U S A. 2008;105: 19114–9. 10.1073/pnas.0801737105 19060190 PMC2614724

[48] Naidoo R, Du Preez P, Stuart-Hill G, Jago M, Wegmann M. Home on the range: factors explaining partial migration of African buffalo in a tropical environment. PLoS One. 2012;7: e36527 10.1371/journal.pone.0036527 22570722 PMC3343005

[49] Lundberg J, Moberg F. Mobile Link Organisms and Ecosystem Functioning: Implications for Ecosystem Resilience and Management. Ecosystems. 2003;6: 0087–0098. 10.1007/s10021-002-0150-4

[50] Bull JW, Suttle KB, Singh NJ, Milner-Gulland E. Conservation when nothing stands still: moving targets and biodiversity offsets. Front Ecol Environ. 2013;11: 203–210. 10.1890/120020

[51] Singh NJ, Milner-Gulland EJ. Conserving a moving target: Planning protection for a migratory species as its distribution changes. J Appl Ecol. 2011;48: 35–46. 10.1111/j.1365-2664.2010.01905.x

